# Problem-based learning versus reflective practice on nursing students’ moral sensitivity

**DOI:** 10.1186/s12912-023-01377-8

**Published:** 2023-06-20

**Authors:** Toktam Zia, Hakimeh Sabeghi, Gholamhossein Mahmoudirad

**Affiliations:** 1 Faculty Member, Department of Medical Surgical Nursing, School of Nursing and Midwifery, Birjand University of Medical Sciences, Birjand, Iran; 2 Department of Nursing, School of Nursing and Midwifery, Birjand University of Medical Sciences, Birjand, Iran

**Keywords:** Ethics Education, Moral Sensitivity, Problem-based Learning, Reflective practice, Nursing student, Empirical approaches

## Abstract

**Background:**

Moral sensitivity is one of the prerequisites for nurses’ professional competency and patient care. It is critical to teach professional ethics in a student-centered manner in order to increase students’ moral sensibility. This study evaluated the effects of professional ethics education via problem-based learning and reflective practice on nursing students’ moral sensitivity.

**Methods:**

This experimental study was performed on 74 nursing students who were randomly divided into three groups of problem-based learning, reflective practice and control. Principles of professional ethics were presented for the two intervention groups in four 2-hour sessions using ethical dilemmas scenarios. Participants completed the Moral Sensitivity Questionnaire before, immediately, and three months after the intervention. Data were analyzed using SPSS_16_.

**Results:**

Demographic characteristics of the three groups were similar (p > 0.05). The total moral sensitivity score significantly differed between the groups immediately and three months after the intervention (p < 0.001). The total mean score for moral sensitivity was significantly different between the two groups of problem-based learning and reflective practice, with the mean scores in the problem-based learning group being higher (p = 0.02). The mean score of moral sensitivity decreased statistically significantly in both experimental groups three months after the intervention as compared to immediately after the intervention (p < 0.001).

**Conclusion:**

Nursing students’ moral sensitivity can be increased through reflective practice and problem-based learning. While the results indicated that problem-based learning was more successful than reflective practice, additional research is recommended to confirm the influence of these two strategies on moral sensitivity.

## Background

Moral sensitivity is one of the prerequisites for nurses’ professional competency and humane principles of patient care [1]. Nurses face a variety of moral dilemmas as a result of their close interaction with the patients [2]. Therefore, understanding the codes of ethics is insufficient; nurses must also possess the appropriate value resources in order to practice ethically, which results from sensitivity to ethical principles [3].

With moral sensitivity as a critical component of ethics, nurses are able to recognize ethical issues in their professional environment, provide effective and ethical care to patients, and make moral decisions [4]. According to Lutzen et al., moral decision-making consists of four components: moral sensitivity, moral judgment, moral motivation, and moral character [5]. Based on the results of several studies, the most critical component of moral decision making is moral sensitivity, which helps nurses to recognize ethical issues while delivering patient care and hence make more informed and right decisions when confronted with moral dilemmas [6–9]. Moral sensitivity is a personal characteristic and a fundamental need for nurses to recognize, interpret, and respond to ethical issues involving patients, as well as the potential influence of their practices on patients’ health [10]. Nurse’s strong moral sensitivity and perception of professional roles and responsibilities in ethical circumstances result in an increase in the quality of care and the health of patients [5, 11].

Nursing students must also possess a high level of moral sensitivity, since they will be exposed to high-risk ethical situations in their workplace and will be responsible for providing comprehensive care to patients based on ethical decision-making skills [12]. Existing research indicates that nursing students have a lower level of moral sensitivity than nurses [13, 14], which can result in a sense of weakness when confronted with ethical dilemmas in clinical settings [15]. As a result, nursing students must be equipped to deal with ethical dilemmas in the future [16–19]. Nursing students require some form of instruction included in the curriculum that enables them to comprehend and apply ethical principles and issues during their patient care [20]. By incorporating ethical concepts, codes, and reflection into the nursing curriculum, nursing students can become aware of ethical dilemmas in clinical settings [17].

Teaching methods of ethics in universities are frequently based on theory and lack a strong connection to clinical practice [21]. The most frequently used teaching method of ethics is lecture, although existing evidence indicates that this strategy results in passive learning [21, 22]. Evidence show that, traditional teaching methods, which rely on principles, laws, theories, and codes of ethics, do not always prepare nurses for ethical decision-making in the clinic, and they are insufficient to improve nurses’ ethical decision-making ability [23, 24]. As a result, using student-centered and active learning approaches in nursing education programs for teaching ethical principles is essential [25].

Problem-based learning is a student-centered teaching method that emphasis on group discussion. It enables students to develop essential skills such as personal communication, critical thinking, decision-making, reasoning, teamwork, cooperation, respect for team members, curiosity, and tolerance in addition to providing in depth learning [26, 27].As Jarvis says, “reflective practice is something more than a thoughtful practice, which seeks to problematize many situations of professional performance so that they can become potential learning situations and so the practitioners can continue to learn, grow and develop in and through their practice” [28]. Reflective learning not only results in the development of knowledge and skills, but also bridges theory and practice, which serves as the foundation for evidence-based, practice [29]. In ethics education, reflective practice enables students to comprehend the nature of moral dilemmas and their interrelationships [30].

The application of innovative and active learning strategies in ethics necessitates educational research. To determine which method is more effective in terms of moral sensitivity, the researchers compared and evaluated the effect of professional ethics education via reflective practice and problem-based learning on the moral sensitivity of nursing students.

## Method

### Design

This randomized controlled experimental study was performed on undergraduate nursing students of Birjand University of Medical Sciences, Iran.

### Eligibility criteria

The study included third- and fourth-year nursing students who expressed a desire and satisfaction to participate in the study and had no prior work experience in either of the health care systems. Exclusion criteria were students who did not wish to continue the intervention for any reason during the study, or those who attended only once in training sessions.

### Variables and instruments

The Moral Sensitivity Questionnaire (MSQ) developed by Lutzen (1994) was used to examine students’ moral sensitivity [31]. Then it has been used in different countries including Iran [32]. This questionnaire is divided into two sections. The first section contains demographic data. The second section has 25 questions that assess nurses’ ethical decision-making when providing clinical care. Each question is assessed on a five-point Likert scale: strongly agree (4), somewhat agree (3), somewhat disagree (2), strongly disagree (1), and have no idea (0).on a five-point Likert scale from strongly agree to strongly disagree This questionnaire measures moral sensitivity on six dimensions, including the following: 1- modifying autonomy 2-interpersonal orientation, 3-trust in medical knowledge and principles of care, 4- experiencing moral conflict, 5- structuring moral meaning, 6-benevolence. The maximum score is 100, while the minimum score is zero. Accordingly, a total score of 0–50 indicates poor moral sensitivity, 50–75 indicates moderate moral sensitivity, and 75–100 indicates great moral sensitivity [32]. The questionnaire’s validity and reliability have been confirmed in earlier studies (Cronbach’s alpha: 80%) [32].

### Data collection and procedures

74 undergraduate nursing students in 2019 and 2020 academic years (the 5th and 7th semester) were divided into 12 clinical groups, each group contained an average of 6–7 students. Then these groups were divided into three groups (problem-based learning, reflective practice, and control) using the permuted block technique. All students were randomly assigned to groups if they had informed consent and had no previous clinical work experience. Before the intervention, all students completed the moral sensitivity questionnaire.

Four 2-hour sessions of educational interventions were held. Two nursing professors approved the educational content, which included professional ethics in nursing, nursing codes of ethics, patient rights, ethical decision-making, and professional communication. The educational content was prepared as an educational package (which included a concept map and pamphlet). Additionally, six classic moral dilemma scenarios [33] were applied.

For the problem-based learning group (PBL) the researcher explained the course objectives, the students’ responsibilities, and the problem-solving approach at the first session using PowerPoint software. The students were then broken into smaller groups of three to four individuals and given the educational package. Two of the six moral dilemma scenarios were given to the groups throughout the second to fourth sessions. They were instructed to discuss and document the following seven PBL steps for each dilemma: 1- Definition of concepts, 2- defining the problem, 3- discussing/analyzing the problem, 4- identifying possible solutions, 5- setting objectives and prioritizing the problem, 6- problem solutions, and 7- problem-solving based on the measures.

The first session for the reflective group was identical to that for the PBL group. Students were instructed on how to conduct reflections and the stages involved. Between the second and fourth sessions, two of the six moral scenarios were allocated to each group, and they were invited to discuss and provide comments on a reflective practice based on Atkins and Murphy’s theory. This structure is comprised of five distinct stages [34]:

#### Self-awareness

it entails being conscious of one’s discomforts regarding the scenario.

#### Description

it includes thoughts and feelings, the key points and characteristics (pros and cons).

#### Analysis

it examines the components of a situation in order to identify current knowledge and hypotheses, as well as to challenge, imagine and explore alternative solutions.

#### Integration

it is associated with a shift in perspective. This stage may result in emotional and cognitive alterations in thinking. At this step, prior knowledge is combined with new knowledge, and creativity is used to address the problem and a new perspective is created.

#### Evaluation

a decision is made on the worth of something, which frequently entails criteria and standards.

The researcher was present at the small group discussion sessions, so students could seek assistance from the instructor at any time. At the end of each session, participants shared their opinions with the whole group, asked their questions and resolved their ambiguities regarding moral dilemmas.

Each scenario took an average of 15–20 min for participants in both intervention groups. After completing the procedures, the groups discussed the scenario, and then, students were asked to describe similar situations they encountered and how they handled them.

The control group did not receive any instruction until the end of intervention. To ensure compliance with ethical issues, these students were presented with an educational booklet and ethical scenarios at the end of the study. Students in three groups recompleted the Moral Sensitivity Questionnaire immediately and three months after the intervention.

### Ethical considerations

After obtaining the code of ethics (Ir.bums.REC.1398.212) from the ethics committee of the University of Medical Sciences in Eastern Iran, the objectives of the study, the duties of the students and the role of the researcher were clearly explained to the participants and their informed written consent was obtained. The students were assured that the information obtained from them would be kept confidential and that they could withdraw from the study at any stage of the study.

### Data analysis

Using SPSS for Windows 11.5 (SPSS Inc., Chicago, IL, USA), the collected data were analyzed by descriptive statistics as well as Chi-square, ANOVA, Mann-Whitney, repeated measures ANOVA and Kruskal-Wallis tests.

## Results

### Participants’ socio-demographics

The mean ages of problem-based learning, reflective practice and control groups were 21.44 ± 0.87, 21.52 ± 1.08 and 21.71 ± 0.90, respectively, which did not show a statistically significant difference (P = 0.60). There was no statistically significant difference in the mean grade average between the three groups of problem-based learning (16.56 ± 0.97), reflective practice (16.32 ± 1.0) and control (16.69 ± 1.05) (P = 0.42). In addition, there was no significant difference in age, sex, semester, marital status and history of participation in the ethics seminar between the three groups (P > 0.05) (Table 1).

**Table 1:** Demographic groups of students Reflective and PBL and Control (n = 74)

Socio-demographic characteristics		PBL group	Reflective group	Control group	Chi-square statistic test results (p value
Gender	Man Female	8 (32%) 17 (68%)	11 (44%) 14 (56%)	12 (50%) 12 (50%)	X^2^ = 1.69 P = 0.42
semester	Term 5 Term 7	13 (52%) 12 (48%)	10 (40%) 15 (60%)	12 (50%) 12 (50%)	X^2^ = 0.82 P = 0.66
Marital status	Single Married	20 (80%) 5 (205)	23 (92%) 2 (8%)	19 (79%) 5 (21%)	X^2^ = 1.88 P = 0.39
history of participation in the ethics seminar	Yes No	21 (84%) 4 (16%)	19 (76%) 6 (24%)	18 (75%) 6 (25%)	X^2^ = 0.71 P = 0.70

### Outcomes

Except for autonomy (P = 0.02), the one-way analysis of variance revealed no statistically significant difference in the mean scores of moral sensitivity and its dimensions between the groups of problem-based learning and reflective practice and control before the intervention. The mean scores of moral sensitivity and its dimensions improved considerably in PBL and reflective practice groups immediately and three months after the intervention (P < 0.05), with the PBL group improving more than the reflective practice group. The mean scores of moral sensitivity and its dimensions did not increase in control group immediately and three months after the intervention (P > 0.05) (Table 2).

**Table 2:** Comparison of the mean scores of total moral sensitivity and its dimensions in the three groups before, immediately and three months after the intervention

Dimensions of moral sensitivity		Before (Mean ± SD)	Immediately after (Mean ± SD)	After 3 month of intervention (Mean ± SD)	RM-ANOVA	Two-way of RM-ANOVA
Modifying autonomy	PBL Reflective Control One way ANOVA	6.16 ± 1.37 5.80 ± 1.38 7.00 ± 1.88 0.02	10.00 ± 0.86 9.32 ± 1.34 6.83 ± 1.60 < 0.001	9.60 ± 1.04 8.32 ± 1.28 6.91 ± 1.63 < 0.001	P < 0.001 P = 0.75 P < 0.001	P < 0.001
Interpersonal orientation	PBL Reflective Control One way ANOVA	13.52 ± 1.50 13.32 ± 2.39 12.79 ± 2.73 0.51	18.64 ± 1.03 18.00 ± 1.73 12.87 ± 2.50 < 0.001	17.64 ± 1.28 16.16 ± 1.88 12.66 ± 2.33 < 0.001	P < 0.001 P = 0.75 P < 0.001	P < 0.001
Trust in medical knowledge and principles of care	PBL Reflective Control One way ANOVA	3.80 ± 1.41 3.40 ± 1.73 4.20 ± 1.74 0.23	6.44 ± 1.00 5.56 ± 1.32 4.29 ± 1.42 < 0.001	6.04 ± 0.97 4.80 ± 1.37 4.54 ± 1.55 < 0.001	P < 0.001 P = 0.19 P < 0.001	P < 0.001
Experiencing moral conflict	PBL Reflective Control One way ANOVA	7.40 ± 1.60 6.28 ± 2.22 6.45 ± 1.86 0.09	10.80 ± 1.19 10.12 ± 1.56 6.83 ± 1.57 < 0.001	10.28 ± 1.02 8.84 ± 1.54 6.70 ± 1.65 < 0.001	P < 0.001 P = 0.21 P < 0.001	P < 0.001
Structuring moral meaning	PBL Reflective Control One way ANOVA	9.84 ± 1.90 10.88 ± 1.83 9.75 ± 2.43 0.11	16.64 ± 1.49 16.64 ± 1.31 9.70 ± 2.33 < 0.001	15.64 ± 1.55 14.68 ± 1.51 10.00 ± 2.41 0.001	P < 0.001 P = 0.40 P < 0.001	P < 0.001
Benevolence	PBL Reflective Control One way ANOVA	14.76 ± 2.89 14.48 ± 3.07 14.91 ± 3.30 0.88	23.72 ± 2.11 22.44 ± 2.43 15.04 ± 3.05 < 0.001	22.88 ± 2.20 19.88 ± 2.68 14.58 ± 3.04 < 0.001	P < 0.001 P = 0.23 P < 0.001	P < 0.001
Total	PBL Reflective Control One way ANOVA	55.48 ± 4.10 54.16 ± 4.80 55.12 ± 6.46 0.65	86.24 ± 4.16 82.08 ± 6.63 55.58 ± 5.19 < 0.001	82.08 ± 4.38 72.72 ± 6.05 55.41 ± 5.24 < 0.001	P < 0.001 P = 0.73 P < 0.001	P < 0.001

RM-ANOVA: repeated measures analysis of variance; SD: standard deviation; PBL: problem-based learning

When mean scores of moral sensitivity were compared, a two-way repeated measures ANOVA test revealed a statistically significant difference in total score of moral sensitivity and its dimensions scores between the three groups (P < 0.05) (Table 2). The Bonferroni test showed a significant difference in the total score of moral sensitivity and its dimensions scores between the PBL and control groups (P < 0.05). When the two groups of reflective practice and control were compared, a significant difference was observed in all dimensions except professional knowledge (P < 0.05). There was no significant difference in all dimensions except moral conflicts between PBL and reflective practice groups (P > 0.05). There was, however, a significant difference in the total mean scores of moral sensitivity (P < 0.05) (Table 3).

**Table 3:** Comparison of groups with Bonferroni correction

Dimensions of moral sensitivity		Mean difference	Std. Error	P value
Modifying autonomy	PBL vs. control Reflective vs. control PBL vs. reflective	1.67 0.89 0.77	0.35 0.35 0.34	< 0.001 0.04 0.09
Interpersonal orientation	PBL vs. control Reflective vs. control PBL vs. reflective	3.82 3.04 0.77	0.52 0.52 0.51	< 0.001 < 0.001 0.41
Trust in medical knowledge and principles of care	PBL vs. control Reflective vs. control PBL vs. reflective	1.07 0.25 0.82	0.36 0.36 0.36	0.01 1.00 0.07
Experiencing moral conflict	PBL vs. control Reflective vs. control PBL vs. reflective	2.82 1.74 1.08	0.4 0.4 0.4	< 0.001 < 0.001 0.02
Structuring moral meaning	PBL vs. control Reflective vs. control PBL vs. reflective	4.22 4.24 -0.02	0.48 0.48 0.47	< 0.001 < 0.001 1.00
Benevolence	PBL vs. control Reflective vs. control PBL vs. reflective	5.60 4.08 1.52	0.74 0.74 0.73	< 0.001 < 0.001 0.12
Total	PBL vs. control Reflective vs. control PBL vs. reflective	19.22 14.27 4.94	1.34 1.34 1.33	< 0.001 < 0.001 < 0.001

PBL: problem-based learning

There was a significant difference in the mean different of total score of moral sensitivity and its dimensions scores between the three groups (P < 0.05). The mean difference of total score of moral sensitivity and its dimensions immediately after the intervention compared to before the intervention is higher than the mean difference in scores three months after the intervention compared to before the intervention. This suggests that the total score for moral sensitivity and its dimensions scores reduced after three months (Table 4).

**Table 4:** Comparison of mean variances of total moral sensitivity and its dimensions in nursing students, immediately and three months after the intervention compared to before the intervention in three groups

Dimensions of moral sensitivity		Immediately compared to before (Mean variances ± SD)	Three months later compared to before the (Mean variances ± SD)	Three months later compared to immediately after (Mean variances ± SD)
Modifying autonomy	problem-based learning Reflective Control Kruskal-Wallis H test	3.84 ± 1.31 3.52 ± 1.26 -0.16 ± 1.04 < 0.001	3.44 ± 1.41 2.52 ± 1.44 -0.08 ± 1.10 < 0.001	-0.40 ± 0.70 -1 ± 0.86 0.08 ± 1.10 < 0.001
Interpersonal orientation	problem-based learning Reflective Control Kruskal-Wallis H test	5.12 ± 1.33 4.68 ± 1.95 0.08 ± 1.34 < 0.001	4.12 ± 1.50 2.82 ± 1.77 -0.12 ± 1.45 < 0.001	-1.00 ± 1.04 -1.84 ± 0.94 0.20 ± 1.28 < 0.001
Trust in medical knowledge and principles of care	problem-based learning Reflective Control Kruskal-Wallis H test	2.64 ± 0.86 2.16 ± 1.74 0.08 ± 0.77 < 0.001	2.24 ± 0.92 1.44 ± 1.26 0.33 ± 1.09 < 0.001	-0.40 ± 0.64 -0.72 ± 0.89 0.25 ± 0.89 < 0.001
Experiencing moral conflict	problem-based learning Reflective Control Kruskal-Wallis H test	3.40 ± 1.65 3.84 ± 1.81 0.37 ± 0.96 < 0.001	2.88 ± 1.45 2.56 ± 1.66 0.25 ± 1.22 < 0.001	-0.52 ± 0.82 -1.28 ± 0.93 -0.12 ± 0.94 < 0.001
Structuring moral meaning	problem-based learning Reflective Control Kruskal-Wallis H test	6.80 ± 1.55 5.76 ± 2.16 -0.04 ± 1.16 < 0.001	5.80 ± 1.52 3.80 ± 1.93 0.25 ± 1.11 < 0.001	-1.00 ± 1.25 -1.96 ± 1.24 0.29 ± 1.12 0.001
Benevolence	problem-based learning Reflective Control Kruskal-Wallis H test	8.96 ± 1.33 7.96 ± 2.58 0.12 ± 1.26 < 0.001	8.12 ± 1.71 5.40 ± 2.39 -0.33 ± 1.40 < 0.001	-0.84 ± 0.94 -2.56 ± 1.58 -0.45 ± 1.35 < 0.001
Total	problem-based learning Reflective Control Kruskal-Wallis H test	30.76 ± 3.03 27.92 ± 7.56 0.45 ± 3.24 < 0.001	26.60 ± 3.50 18.56 ± 6.18 0.29 ± 2.92 < 0.001	-4.16 ± 2.39 -9.36 ± 3.13 -0.16 ± 2.31 < 0.001

SD: standard deviation

## Discussion

The results of this study indicated a rise in the mean scores of moral sensitivity of PBL students across all stages. Mean moral sensitivity scores had a substantial increase in the PBL group across all stages (P < 0.01). As a result, problem-based ethics education can help develop moral sensitivity. In comparison to many studies which show that PBL is an efficient way for ethics education and can help nursing students to acquire professional competency [35–38], Yeom (2017) demonstrated that teaching ethics via lectures, group discussions, and questions and answers had no effect on students’ total moral sensitivity scores [4]. Carrero et al. also found no difference in participants’ knowledge between problem-based learning and lecture-based groups [39]. Similarly, the results of a study in the Netherlands showed When PBL and traditional learning methods were compared among nursing students, no significant differences in general and clinical competencies, as well as professional development, were discovered [40].

One of the reasons for these distinctions could be the way ethics education and problem-based training are delivered. According to prior research, achieving learning objectives requires an appropriate PBL design, coaching, and a framework to promote individual learning [41, 42]. Holding such courses involves time and supervision is challenging. However, in the current study, educational courses were held small group format with discussion sessions and in the presence of a mentor.

At all stages of the study, data analysis revealed an increase in the total mean score of moral sensitivity and its dimensions scores in the reflective practice group. Previous researches showed that ethical reflection improved health care workers’ self-confidence, ability to solve problems, and moral awareness [43, 44]. Contras (2020) demonstrated that reflective practices had a positive effect on undergraduate nursing students. In nursing practice, reflective approaches minimize stress and anxiety while increasing learning, competency, and self-awareness [45]. Another study in 2018 also showed that reflection enhanced students’ competency to participate fully in clinical practice [46]. As previously noted, reflection entails reviewing one’s beliefs and attitudes critically in order to develop self-awareness, self-monitoring, and self-regulation (Mann et al., 2009). It is a means of bridging the gap between idea and action, as well as a means of describing internal processes, evaluating obstacles, and identifying accomplishments [47].

Teaching method had an effect on students’ moral decision-making skills and produced variable results [48]. The use of student-centered strategies for lived clinical practice experience is suggested in ethics education [49].

Although the results of reflective method were nearly identical to PBL, we also found PBL to result in higher mean scores compared to reflective practice.

Although the total score of moral sensitivity and its dimensions scores showed a significant difference in both PBL and reflective practice groups three months after the intervention, they declined compared to immediately after the intervention. This conveys an essential message that ethical lessons alone are not enough, and in order to sustain the effect of this instruction, it is critical to adhere to the teachings presented. Weshel stressed that ethics is a fluid discipline, and learning it all at once is insufficient and repetition and practice of what has been learned are essential [50]. Several studies, such as Gallager and Choudin’s review studies, as well as Yarbrook and Klotz’s study, indicate that continuing education is required to preserve the efficacy of ethics education [2, 51].

## Conclusion

According to Rest et al. “no one has yet developed an adequate map of the entire moral universe.” [52] However, those of us committed to providing good or moral care must seek out the most effective therapies for promoting ethics and increasing moral sensitivities. This study demonstrated that problem-based learning and reflective practice in ethics education could help improve nursing students’ moral sensitivity. As a result, the researchers recommend using PBL and reflective methods to teach ethics to nursing students. Additional research with larger sample sizes and longer time is necessary to confirm the influence of these two techniques on moral sensitivity. Additionally, it is recommended to conduct research on these methods with other medical groups and centers.

### Limitations

Among the limitations of the study is the selection of participants from only one nursing school. This is because information might have been exchanged between the groups, although the groups were asked not to share their observations with the other groups in any case.

## Data Availability

The datasets used and/or analyzed during the current study are available from the corresponding author upon reasonable request.

## Abbreviations

PBL: Problem-based learning
MSQ: The Moral Sensitivity Questionnaire
SPSS: Statistical Package for the Social Sciences
